# A multidimensional analysis of generative AI adoption in academia: synthesizing TAM3 and UTAUT2 frameworks to predict students’ attitudes and learning effectiveness

**DOI:** 10.3389/frai.2026.1897946

**Published:** 2026-10-07

**Authors:** Golan Carmi

**Affiliations:** Department of Information Systems, Jerusalem College of Technology, Jerusalem, Israel

**Keywords:** ethical knowledge, gender, generative AI, learning effectiveness, students’ attitudes, students’ experience, TAM3, training

## Abstract

The rapid integration of generative artificial intelligence (GenAI) tools into higher education raises important questions regarding their impact on student learning. Based on established technology acceptance frameworks, this study develops and empirically tests an integrative model that explains students’ attitudes toward GenAI tools and their perceived learning effectiveness. Data were collected from 566 undergraduate students using a structured questionnaire. The main findings indicate that prior experience, perceived merits, and training in using GenAI tools are positively associated with and constitute strong predictors of both attitudes of students toward GenAI use and perceived learning effectiveness, while ethical knowledge and technological orientation do not emerge as significant predictors. Perceived drawbacks are also significant, suggesting that ambivalence and concerns about GenAI can diminish students’ positive attitudes and reduce their perceived usefulness. Differences across gender further suggest that technology adoption processes are not uniform but contingent upon user characteristics. Theoretically, this study contributes to the literature by extending technology acceptance models into the domain of GenAI and by linking attitudinal constructs with learning-related outcomes within a unified framework. Practically, the findings suggest that higher education institutions should not only provide access to GenAI tools but also complement this access with pedagogical support, including structured training, comprehensive ethical guidance, and the development of students’ GenAI literacy. Overall, the study provides a nuanced understanding of how GenAI tools are adopted and leveraged in academic contexts and offers a foundation for future research on GenAI-enabled learning.

## 1 Introduction

The advent of generative artificial intelligence (GenAI) in academia marks a profound technological shift, reshaping learning processes and redefining traditional teaching practices. The unprecedented accessibility and availability of large language models (LLMs) enable students to streamline complex tasks, including writing code, analyzing data, transforming information, and producing high-level academic texts. As these technologies become increasingly intuitive, they are rapidly emerging as integral components of the cognitive toolbox of the 21st-century learner.

From a developmental perspective, while traditional AI systems have primarily focused on analyzing existing data and predicting trends, GenAI technologies exhibit unprecedented capabilities for generating original content, including textual, auditory, and visual outputs (Espinoza Vidaurre et al., 2024). The integration of GenAI technologies across workplaces, educational institutions, and everyday contexts raises substantial social, economic, and occupational questions that require careful examination (Wang et al., 2024). The ability to generate images, videos, music, and presentations through simple natural-language prompts using highly advanced GenAI systems within a short timeframe represents a major milestone in digital technology and human–computer interaction (Wen et al., 2025).

GenAI technologies hold considerable potential for enhancing academic learning, teaching, and research processes while simultaneously introducing significant technological, pedagogical, and ethical challenges for both instructors and students (Floridi, 2023; Katsamakas et al., 2024). The rapid evolution of GenAI tools creates a dynamic technological landscape. Such developments enable personalized learning and improve academic efficiency. However, their effective adoption depends on a complex interplay of social, psychological, and technological factors within the academic environment (Xia et al., 2024).

Social dynamics play a central role in shaping the adoption of GenAI technologies, with subjective norms and peer influence affecting students’ perceptions regarding the legitimacy and appropriateness of using such tools. At the individual level, factors such as technological self-efficacy and GenAI-related anxiety contribute to emerging forms of digital inequality: while some students rapidly adopt these tools as cognitive and creative partners, others experience uncertainty or resistance in response to rapid technological changes. These factors collectively influence teaching and learning processes in higher education institutions, which are required to respond effectively to this evolving techno-pedagogical phenomenon (Olawale and Mutongoza, 2024; Wu et al., 2025). In addition, the integration of GenAI tools into academic contexts raises significant ethical concerns related to responsible use, academic integrity, and transparency. These concerns extend beyond issues of plagiarism to encompass broader questions regarding the development of critical thinking skills and the preservation of students’ intellectual autonomy (Dabis and Csáki, 2024). A persistent tension exists between the efficiency gains afforded by GenAI tools and the need to ensure accuracy, transparency, and the mitigation of algorithmic and informational biases (Zhang and Qian, 2024).

Since the introduction of GenAI tools into academia, a growing body of research has examined their adoption and use among students. Existing studies consistently report rapid uptake of these technologies across diverse academic disciplines, alongside their increasing integration into students’ learning practices (Chan and Hu, 2023; Crompton and Burke, 2023; Duy et al., 2025; Elchaghaby and Wahby, 2025; Fan et al., 2025; Gasaymeh et al., 2024; Sáez-Velasco et al., 2025; Sami et al., 2025; Türk et al., 2025; Vieriu and Petrea, 2025). Despite this growing body of literature, several important gaps remain. First, prior research has largely focused on descriptive patterns of use and general attitudes toward GenAI, with limited attention to the complex interplay between technological, psychological, and social determinants, referring only to ChatGPT at times. Second, there is a lack of integrative frameworks that simultaneously account for both enabling factors and inhibiting factors regarding GenAI as a cognitively disruptive technology. Third, empirical evidence regarding how these factors jointly influence learning-related outcomes remains fragmented and inconclusive. To address these gaps, the present study is grounded in established technology adoption frameworks, particularly the Technology Acceptance Model (TAM) and its extended variants, as well as the latest version of Unified Theory of Acceptance and Use of Technology (UTAUT). These theoretical perspectives provide a robust foundation for examining how cognitive, social, and technological factors shape the adoption and use of GenAI tools in academic contexts.

Accordingly, the primary objective of this study is to examine the factors influencing students’ adoption of GenAI tools and to investigate how such factors shape their attitudes, behavioral intentions, and perceived learning effectiveness. This study contributes to the literature in three key ways. First, it develops an integrative conceptual model that combines technological, psychological, and social determinants of GenAI adoption. Second, it empirically examines the simultaneous effects of enabling and inhibiting factors on students’ attitudes and learning-related outcomes. Third, it extends established technology acceptance frameworks to the emerging context of GenAI in higher education.

The remainder of this paper is structured as follows. First, a literature review is presented, outlining the theoretical foundations and relevant empirical studies. Next, the research hypotheses, methodology, and data analysis are described. The discussion section interprets the findings in relation to existing research. Finally, conclusions, recommendations, implications, limitations, and directions for future research are presented.

## 2 Literature review

### 2.1 Theoretical frameworks

#### 2.1.1 TAM3 model

The Technology Acceptance Model (TAM) developed by Davis (1989), is one of the most central and common models in the study of the adoption and acceptance of information technologies. The model is theoretically based on the social psychological model Theory of Reasoned Action (TRA) developed by Fishbein and Ajzen (1975), and its goal is to explain and predict users’ willingness to adopt or reject a new technology. The model offers a simple, parsimonious framework with high predictive validity, and has, therefore, served as a theoretical basis for many studies in various fields, including business administration, information systems, medicine, and education. In the TAM, two perceptual variables determine whether a person will adopt a particular technology: the first variable is Perceived Usefulness (PU) that is, the extent to which the person believes that using the specific technology will improve their performance or efficiency. The second variable is Perceived Ease of Use (PEOU) that is, the extent to which the person believes that using the technology does not require significant physical or mental effort. These two variables influence the following: attitude toward use, behavioral intention to use, and actual use.

Over the years, more advanced versions of the initial TAM have been developed. The TAM2 developed by Venkatesh and Davis (2000) added two clusters of external variables to the initial model that mainly influence Perceived Usefulness (PU). The first cluster includes social influence processes, which include Subjective Norm (i.e., the perception of social pressure to use the technology), Image (i.e., the extent to which the use of the technology improves the user’s social status), and Voluntariness of use (i.e., whether the use is forced or voluntary). The second cluster includes cognitive-instrumental processes, which include Job Relevance, Output Quality, and Result Demonstrability. TAM2 primarily expands the perception of usefulness instead of that of ease of use and has been used for intra-organizational studies in which normative pressures and hierarchical structures exist. Later on, another extended version called TAM3 was developed by Venkatesh and Bala (2008), which combines the variables added in TAM2 with those that affect the perception of ease of use. The additional variables in the model are divided into two types: (1) Anchors (i.e., general pre-existing beliefs), which include computer self-efficacy, computer anxiety, perceptions of external control, and computer playfulness. (2) Adjustments (i.e., beliefs that develop with experience), which include perceived enjoyment and objective usability. This model offers a comprehensive explanation of both social and cognitive factors and incorporates the influence of the user’s cumulative experience.

#### 2.1.2 UTAUT2 model

Another theoretical model that deals with technology adoption is UTAUT (Unified Theory of Acceptance and Use of Technology) developed by Venkatesh et al. (2003) in order to combine several central theoretical models in the field into a unified integrative model with high predictive ability of intentions to use and actual use of information systems. The UTAUT model is a direct and generalized continuation of the TAM; it includes the core of the TAM, extends it to social and organizational factors, and adds contextual mediating variables. The model includes four core factors that influence the intention to use and actual use. First, Performance Expectancy: The extent to which the user believes that using the technology will provide benefit and improve their achievements (this is the strongest variable in predicting intention to use). Second, Effort Expectancy: The degree of perceived ease and convenience of using the technology. Third, Social Influence: The degree of influence of significant people (managers, colleagues, family, social environment, etc.) on the user’s decision to use the technology. Fourth, Facilitating Conditions: The user’s perception of the existence of resources, knowledge, technical support, and infrastructure that enable the effective use of the technology. This model also includes mediating variables that affect the strength of its relationships: gender, age, experience, and voluntariness of use. These variables sharpen the understanding that adopting technology depends on user characteristics within the social context.

An extended version of the UTAUT model is UTAUT2 developed by Venkatesh et al. (2012) to adjust the model to consumer contexts, not only organizational, and to better explain the adoption and use of technologies among private users. While UTAUT was developed primarily for organizational information systems whose use is sometimes dictated by the organization, the UTAUT2 model focuses more on common technologies for the general public such as mobile applications, social networks, e-commerce, learning technologies, and digital services. The main variables in the UTAUT2 model include the four core variables in the UTAUT model as detailed above, in addition to three new variables relevant to the consumer context. The first new variable is Hedonic Motivation: The degree of enjoyment or satisfaction that the user derives from using the technology. This variable reflects intrinsic motivation and is a key factor in adopting consumer technologies. The second new variable is Price Value: The user’s assessment of the relationship between the perceived benefit of the technology and the financial cost involved in using it. When the perceived benefit is higher than the cost, the price value is positive and supports adoption. The third new variable is habit: The extent to which the behavior is automatic as a result of previous experience. Habit affects both intentions to use and actual use. In this model, all the mediating variables are the same, excluding voluntariness.

The UTAUT2 model is the most advanced stage in the evolution of the TAM. It expands the understanding of technology adoption variables beyond efficiency considerations alone and integrates user experience, habits, and economic considerations; it combines a utilitarian approach with an experiential approach to technology adoption. UTAUT2 is a comprehensive and up-to-date model for examining technology adoption by users, as it combines rational, emotional, and behavioral considerations and provides a strong theoretical framework for research dealing with new digital technologies.

This study utilizes a hybrid theoretical framework based on the evolution of acceptance models, specifically merging TAM3 and UTAUT2. This comprehensive alignment allows for the identification of multidimensional factors influencing students’ intentions and actual usage of GenAI tools.

### 2.2 Students’ attitudes and learning effectiveness using GenAI tools

The rapid diffusion of GenAI technologies in higher education has intensified scholarly interest in understanding students’ attitudes toward these tools and the determinants shaping their acceptance. Attitudes toward educational technologies have long been recognized as a critical predictor of behavioral intention and actual use. In technology acceptance research, attitudes represent an individual’s overall evaluative judgment regarding the use of a particular technology, reflecting cognitive beliefs about its usefulness and affective reactions toward its use. Within higher education contexts, positive student attitudes toward digital learning technologies have been consistently associated with stronger behavioral intentions, higher engagement, and improved learning outcomes. Two theoretical frameworks have been highly influential in explaining students’ attitudes toward emerging technologies: the TAM and its extended variants (i.e., TAM3) and the UTAUT2 model. These models conceptualize technology acceptance as a process shaped by cognitive evaluations of technology attributes and social-contextual influences. Both frameworks have been widely applied in recent empirical studies examining the adoption of GenAI tools among students in higher education.

The TAM and its extensions emphasize the role of PU, PEOU, and related cognitive determinants in shaping users’ attitudes toward technology. TAM3 further incorporates variables such as computer self-efficacy, perceived enjoyment, and experience, providing a more comprehensive explanation of technology acceptance in learning environments. Recent empirical research applying TAM3 conducted among 254 university students in teacher education programs in Austria found that perceived performance outcomes from GenAI usage significantly influenced PEOU and PU, which, in turn, affected students’ attitudes toward AI-supported learning environments. The results indicated that when students perceived GenAI tools as improving academic performance and facilitating learning tasks, their attitudes toward the technology became significantly more positive. However, the study also revealed that ethical considerations surrounding GenAI did not directly influence PU or PEOU, suggesting that students’ attitudes were primarily shaped by perceived academic benefits rather than normative concerns (Bećirović et al., 2025). Similarly, Liu et al. (2025) investigated pre-service teachers’ intentions to adopt GenAI tools using an extended TAM framework. Their findings demonstrated that PU and PEOU significantly predicted behavioral intention, while self-efficacy influenced both constructs, indirectly shaping attitudes toward GenAI adoption. These results suggest that students who feel confident in their ability to interact with GenAI systems tend to develop more positive attitudes toward their integration into academic learning environments. Ittefaq et al. (2025) provide further empirical support from their investigation of international students’ adoption of GenAI tools in higher education. The researchers found that perceived learning value and PU significantly influenced students’ attitudes toward GenAI, which in turn mediated the relationship between perceived value and actual technology adoption. These findings underscore the importance of attitudinal constructs as a psychological mechanism linking perceptions of educational value with behavioral intention.

While the TAM focuses primarily on cognitive perceptions of usefulness and ease of use, the UTAUT2 model expands the explanatory framework by incorporating social and contextual factors such as performance expectancy, effort expectancy, social influence, facilitating conditions, hedonic motivation, habit, and price value. In the context of GenAI adoption in higher education, UTAUT2 has been widely applied to analyze the determinants of students’ attitudes and behavioral intentions. In a recent large-scale study conducted among 464 STEM students in Saudi Arabia applied the UTAUT framework to examine predictors of students’ intention to adopt GenAI in academic contexts, found that performance expectancy emerged as the strongest predictor of behavioral intention, followed by social influence and facilitating conditions. These findings show that students develop positive attitudes toward GenAI tools primarily when they perceive clear academic benefits and when their social environment, including peers and instructors, supports the use of such technologies (BinJwair, 2025). Tran and Le (2025), having used a UTAUT-based model to examine the factors influencing IT students’ willingness to use GenAI for learning, provide further evidence. Survey data collected from university students indicated that performance expectancy, effort expectancy, and facilitating conditions significantly predicted students’ willingness to adopt GenAI tools in academic settings. The authors reported that students’ attitudes mediated the relationship between these constructs and behavioral intention, highlighting the central role of attitudinal evaluations in technology adoption processes.

Present studies indicate that students’ technological orientation is significantly shaped by their academic discipline, particularly when comparing technology-oriented (e.g., STEM) and non-technology-oriented programs (BinJwair, 2025; Liu et al., 2025; Tran and Le, 2025). Moreover, meta-analytic evidence suggests that academic discipline functions as a moderating variable that strengthens the relationships between core TAM and UTAUT constructs, particularly by amplifying the effects of self-efficacy and PU on behavioral intention among students with a technological background (Diao et al., 2024). Taken together, these findings highlight that technological orientation is not merely an individual trait but is deeply embedded in the academic context, with disciplinary differences playing a critical role in shaping both the adoption and effective use of GenAI in higher education. Additionally, empirical studies among students in academia indicate gender differences in terms of adoption intention and usage patterns of GenAI tools. Despite inconsistent research findings, some studies have identified significant gender differences, with men being more likely to intend to adopt GenAI, to express positive attitudes, and to perceive its benefits and extent of use (Elnaem et al., 2025; Kinskofer and Tulis, 2025; Maxwell et al., 2025).

The abovementioned findings reinforce the theoretical assumption in both models that students’ attitudes toward emerging educational technologies are primarily shaped by perceptions of academic utility, ease of interaction, and individual capability in using high digital tools. Students are more likely to develop favorable attitudes when they perceive that AI tools enhance learning efficiency, improve academic performance, and support complex cognitive tasks such as writing, analysis, and problem-solving. These findings suggest that students’ attitudes toward GenAI are primarily pragmatic and performance-oriented, reflecting their evaluation of the technology’s contribution to learning effectiveness (Daher and Hussein, 2024; Luo and Tang, 2026; Prohorovs et al., 2026; Serdenia et al., 2025).

Learning effectiveness is a multidimensional construct in educational research, often defined as the degree to which educational experiences produce desirable outcomes in terms of knowledge acquisition, skills development, and long-term learner competence. Understanding learning effectiveness requires the integration of motivation, thinking and cognitive processes, learning strategies and skills, and the benefits that these factors produce in education in general and in academic contexts in particular.

#### 2.2.1 Skills

In educational research, skills refer to the cognitive and procedural competencies that learners develop, including academic writing, analytical reasoning, problem-solving, and information literacy. The rapid integration of GenAI tools in higher education has raised important theoretical and empirical questions regarding their influence on students’ skill development. Emerging research suggests that GenAI systems can function as cognitive scaffolds that support complex academic tasks. For example, structured GenAI-supported feedback has been found to enhance writing quality, idea generation, and revision processes, potentially strengthening procedural and metacognitive skills (Baidoo-Anu and Owusu Ansah, 2023; Zhai, 2023). Similarly, Farrokhnia et al. (2023) argue that GenAI tools can support higher-order skill acquisition when embedded within pedagogically guided learning environments rather than used as autonomous content generators.

Empirical studies have examined how technology acceptance constructs relate to cognitive- and performance-related skills outcomes in GenAI use. Almassaad et al. (2024) surveyed 859 students and found that PU and PEOU, according to the TAM, were significantly associated with students’ frequent use of GenAI for concept clarification and writing tasks. The authors interpreted this as linking technology acceptance perceptions with practical academic skills in academic tasks such as defining concepts and summarizing literature. The study by Salhab and Aboushi (2025) investigated how AI literacy and 21st-century skills influence students’ acceptance of GenAI based on TAM constructs (PU, PEOU, etc.). Their findings show that students with stronger 21st-century skills, such as critical thinking, collaboration, and adaptive learning, exhibit higher acceptance levels of GenAI, as mediated through core TAM variables, suggesting that proficiency in modern competencies amplifies both acceptance and the skilled application of GenAI tools.

Additionally, BinJwair’s (2025) UTAUT model-based study among 464 STEM students in higher education demonstrated that performance expectancy, the belief that using GenAI will enhance their performance, significantly predicted behavioral intention to use these tools. This outcome suggesting that students who expect GenAI to support their academic work, such as problem-solving and research tasks, are more likely to integrate GenAI into skill-challenging activities. Another comprehensive study by Xu et al. (2025) applied the UTAUT model to examine how GenAI acceptance factors influenced 1,190 students’ usage behavior of GenAI. The results show that performance expectancy and behavioral intention significantly predict use behavior, which can be interpreted as students integrating GenAI into academic tasks that involve skill application, such as research, problem-solving, and content creation. This supports the idea that higher performance expectancy, rooted in UTAUT perceptions, relates not only to intention but actual use of GenAI tools that connect to learning skills.

These studies show that students’ acceptance beliefs, usefulness, and expectancy underpin decisions to use GenAI tools in ways that relate to developing or applying academic skills. The findings position the TAM and the UTAUT model not merely as adoption predictors of students but as mechanisms linking GenAI perceptions with actual performance and skill use. However, concerns have also been raised regarding skill erosion because of overreliance on GenAI systems. The cognitive offloading theory suggests that when learners delegate core intellectual processes to external systems, they may reduce their own cognitive engagement (Kasneci et al., 2023). Empirical findings indicate that unregulated GenAI use may weaken independent analytical writing and problem-solving abilities if students substitute rather than complement their own effort (Rudolph et al., 2023). Therefore, the relationship between GenAI use and high-level skills development appears conditional upon instructional design, applied training, learner regulation, and GenAI literacy.

#### 2.2.2 Motivation

Learning motivation refers to the psychological processes that energize, direct, and sustain academic behavior. GenAI technologies that enhance perceived competence, autonomy, or task value can positively influence students’ learning motivation. Recent studies suggest that GenAI tools may enhance motivation by providing immediate feedback, personalized explanations, and interactive dialogue that increases students’ sense of competence and engagement (Tlili et al., 2023; Zhai, 2023). Baidoo-Anu and Owusu Ansah (2023) report that students frequently describe AI-assisted learning as engaging and confidence-building, particularly when tools help students clarify complex topics or reduce cognitive frustration.

Studies provide concrete evidence that motivational factors significantly influence student engagement in GenAI, strengthening the theoretical link between motivation and technology acceptance in academic contexts. Afzaal et al. (2025) operationalized the TAM to investigate intrinsic motivation as a key determinant in students’ behavioral intention to use ChatGPT for academic purposes. The authors found that intrinsic motivation significantly predicted both PU and PEOU, which in turn increased students’ behavioral intention to use ChatGPT for learning activities. This result supports the theoretical mechanism in the TAM whereby motivational drivers shape acceptance beliefs, ultimately leading to greater tool uptake in learning contexts. A study by Sergeeva et al. (2025) applied the UTAUT2 framework to investigate the determinants of higher education students’ adoption of GenAI technologies. In this study, hedonic motivation (i.e., intrinsic enjoyment) a core UTAUT2 factor was found to be a significant positive predictor of students’ intention to use GenAI tools for learning. This suggests that when students experience intrinsic enjoyment and satisfaction from using GenAI, their overall motivation to integrate these systems into academic tasks increases, strengthening adoption behavior. These findings show that students’ internal motivations, such as interest, enjoyment, and curiosity, are not peripheral but are core mediators in the process of technology adoption that enhance motivational engagement with GenAI in higher education.

Simultaneously, scholars caution that GenAI may shift motivation toward extrinsic efficiency goals, such as completing assignments faster, rather than intrinsic mastery (Cotton et al., 2024; Kasneci et al., 2023). If students primarily value GenAI for performance optimization rather than deep understanding, motivational quality may become instrumental instead of self-determined. Thus, while GenAI can increase learning engagement, its influence on the depth and sustainability of academic motivation depends on how students integrate the technology into their learning process (Almassaad et al., 2024; Sami et al., 2025).

#### 2.2.3 Benefits

Benefits of learning with GenAI tools include the tangible benefits that students derive from using these tools in academic contexts. These benefits may include procedural efficiency (e.g., time savings), mental support (e.g., anxiety reduction), and improved self-regulation (e.g., planning and rehearsal support for studying). Moreover, PU refers to the extent to which individuals believe a technology will enhance their performance. This is the most robust predictor in the TAM and the UTAUT model, and recent research consistently demonstrates its centrality in students’ GenAI acceptance.

Empirical studies consistently show that students perceive GenAI as beneficial for idea generation, drafting support, summarization, and time management (Almassaad et al., 2024; Baidoo-Anu and Owusu Ansah, 2023; Zhai, 2023). Other studies indicate that students frequently report productivity-related benefits when using GenAI systems. For example, AI-assisted drafting and summarization tools have been associated with increased efficiency in completing academic tasks and managing workload demands (Dwivedi et al., 2023; Lim et al., 2023). Structural modeling research further confirms that PU significantly predicts the intention to use AI tools in academic settings proactively (Farrokhnia et al., 2023). Similarly, BinJwair’s (2025) UTAUT-based study reported that performance expectancy, conceptually analogous to PU, was the strongest predictor of behavioral intention among STEM students. This outcome underscores how beliefs about GenAI’s capacities to improve academic performance, such as solving complex problems or clarifying coursework, drive both the intention and the actual adoption of GenAI in academic activities.

In addition to efficiency gains, GenAI tools may offer cognitive structuring benefits. Adaptive AI explanations and iterative feedback mechanisms can support knowledge organization and conceptual integration, especially when learners use AI to compare perspectives, refine arguments, or identify knowledge gaps (Farrokhnia et al., 2023; Zhai, 2023). In this sense, benefits extend beyond timesaving to include enhanced clarity and depth comprehension. Affective benefits have also been documented. Another study reports that students perceive GenAI tools as reducing academic stress and performance anxiety, particularly in writing-intensive disciplines (Baidoo-Anu and Owusu Ansah, 2023). Immediate, non-judgmental feedback from GenAI systems may create a psychologically safe space for experimentation and revision, potentially strengthening academic confidence and persistence.

However, scholars caution that perceived benefits must be critically examined. Some research suggests that while students report substantial short-term advantages, especially efficiency and convenience, these benefits may not always translate into long-term learning gains if GenAI use replaces deliberate practice. Additionally, high PU does not necessarily equate to improved learning outcomes. If usefulness is primarily associated with task efficiency rather than conceptual development, students may overestimate learning gains while reducing cognitive effort. Therefore, the construct of benefits should be conceptualized multidimensionally, distinguishing between short-term functional gains and sustained developmental outcomes (Kasneci et al., 2023; Rudolph et al., 2023).

#### 2.2.4 Understanding

Understanding in higher education refers to students’ ability to construct meaningful knowledge representations, integrate new information with prior knowledge, and explain concepts in coherent and transferable ways. Unlike surface-level memorization, conceptual understanding involves deep processing, abstraction, and the ability to apply knowledge across contexts. GenAI technologies have the potential to enhance students’ conceptual understanding when integrated within structured pedagogical frameworks that require explanation, critique, and synthesis. However, without such scaffolding, its contribution to deep understanding may be limited and even negative.

From a constructivist perspective, learning occurs when students actively engage in meaning-making processes. GenAI tools may influence conceptual understanding by providing explanations, examples, analogies, and reformulations that help students reorganize knowledge structures (Zhai, 2023). When students use GenAI dialogically, such as asking for clarification, requesting alternative explanations, or probing reasoning, GenAI can function as a cognitive partner that supports elaboration and knowledge refinement (Farrokhnia et al., 2023).

Empirical studies suggest that GenAI-supported instructional environments may enhance comprehension by delivering immediate, adaptive feedback that targets students’ performance (Baidoo-Anu and Owusu Ansah, 2023). The availability of iterative explanation cycles allows students to test hypotheses, compare interpretations, and deepen understanding through interactive inquiries. In this sense, GenAI can facilitate elaborative rehearsal and conceptual restructuring, processes central to durable learning. Almassaad et al. (2024) also report that students frequently use GenAI tools to clarify concepts and translate academic content, suggesting that students’ acceptance perceptions are tied not only to ease of use or convenience but also to meaning-making and understanding processes in learning contexts.

Other studies also highlight the importance of metacognitive regulation in mediating the relationship between AI use and understanding. Students who monitor, evaluate, and revise AI-generated explanations demonstrate stronger conceptual gains than those who accept outputs uncritically. Therefore, understanding should be conceptualized not merely as exposure to GenAI explanations but as a function of interactive, reflective engagement with GenAI-generated content (Rudolph et al., 2023). Similarly, BinJwair’s (2025) UTAUT findings identified effort expectancy as significant in shaping students’ intention to employ GenAI for academic tasks. High effort expectancy can reduce cognitive load and support deeper engagement with content, which theoretically enhances conceptual understanding when students allocate cognitive resources to meaning construction instead of struggling with tool complexity.

However, scholars emphasize that conceptual understanding requires active cognitive engagement. If students rely on GenAI-generated summaries or explanations without critically processing or reconstructing the content, learning may remain superficial (Kasneci et al., 2023). While using GenAI tools may reduce cognitive load by simplifying explanations, it may also inadvertently reduce germane cognitive load if learners do not engage in effortful processing. Thus, the impact of GenAI on understanding depends on whether students use the tools for active cognitive elaboration or passive consumption of output (Dwivedi et al., 2023).

The body of research reviewed above reflects the rapid proliferation and growing prominence of GenAI tools among students in higher education. This accelerating adoption underscores the need for more theoretically grounded and integrative investigations into the implications of such tools as an embedded component of academic learning processes. While prior studies have generated valuable insights, a substantial proportion of this work has focused on students’ attitudes and perceptions within relatively constrained empirical settings, often limited to a single dominant tool—mostly ChatGPT—or to homogeneous samples drawn from a single disciplinary context. Other studies have relied on only a single TAM or a basic model that is not theoretically up-to-date, and some have examined the technologies relatively soon after their emergence, which limits students’ familiarity and use.

Drawing on advanced theoretical perspectives and using the TAM3 and the UTAUT2 model as a hybrid framework, the present study addresses this gap by adopting a more comprehensive and mechanism-oriented approach. Specifically, it conceptualizes students’ engagement with GenAI tools as a function of interrelated cognitive, experiential, and contextual determinants, extending beyond the traditional emphasis on PU and PEOU. Empirically, the study advances prior research by examining a heterogeneous sample of students across diverse fields of knowledge who engage with diverse GenAI tools, thereby enhancing both ecological validity and generalizability. In addition, it integrates both attitudinal and outcome-based constructs by jointly examining students’ attitudes and perceived learning effectiveness, offering a more holistic account and comprehensive evaluation of educational outcomes associated with GenAI use.

Moreover, the study contributes to the emerging literature by incorporating a broader set of explanatory variables, including training, experience, ethical knowledge, perceived merits and drawbacks, and technological orientation, thereby aligning with and extending the multidimensional structure proposed in contemporary TAMs. Consequently, it not only tests established theoretical relationships but also refines and contextualizes them within the rapidly evolving domain of GenAI in academia. Scientifically, this approach positions the study as a theoretically informed and empirically robust contribution that advances current understanding of how GenAI technologies are adopted, interpreted, and leveraged for learning in higher education contexts. We contend that a continuous examination of students’ engagement with GenAI tools is imperative, given the rapid technological evolution, the iterative nature of these systems, and the growing institutionalization of GenAI tools in higher education. Furthermore, such ongoing scrutiny is essential for capturing the cumulative proficiency and lived experiences of students who integrate these intelligent learning tools into their daily academic routine.

## 3 Research goals

This research is designed to empirically assess students’ GenAI tool utilization. The specific aims are (1) to measure the learning effectiveness of students using GenAI and (2) to analyze their attitudes concerning these tools in academic learning. The theoretical framework of this study employs an integrated approach, combining two of the most robust and contemporary models in information technology research: TAM3 and UTAUT2. While the TAM3 emphasizes the cognitive and psychological factors that shape perceptions of ease of use and usefulness, the UTAUT2 model delves into social and behavioral motivations for technology use. By synthesizing these models, the study captures a broader range of determinants, including individual perceptions of utility, social influence, and facilitating conditions, which together provide a wide perspective for understanding the adoption of advanced technologies in learning environments. Combining these models allows us to examine how students’ characteristics, prior experience, and institutional context predict attitudes and learning effectiveness when using GenAI tools. Based on the theoretical models and the studies presented in the literature review, six research hypotheses were derived.

### 3.1 Academic training on GenAI tools

According to the TAM3, academic training acts as a mechanism of perceptions of external control that is, the perception of the availability of resources and organizational support, which reduces perceived effort and increases the perception of ease of use. In addition, training reduces computer anxiety and strengthens computer self-efficacy—a key predictor of technology adoption. In the UTAUT2 model, Training reflects facilitating conditions, which increase actual use and reduce barriers to adoption. When students feel that a supportive infrastructure exists, the likelihood of developing performance expectancy increases, which is directly related to improved performance, which in the academic context is more effective learning. This can be described as follows: Institutional training → provides facilitating conditions → higher ease of use → higher PU → higher performance expectancy → high positive attitudes and more effective learning. Therefore, the first hypothesis is as follows:

*H1*: Students who have undergone formal institutional training in using GenAI tools will report higher levels of perceived learning effectiveness and more positive attitudes toward the use of these tools.

### 3.2 Experience with GenAI tools

According to the TAM3, cumulative experience is a mechanism of adjustments, in which users develop more realistic perceptions of the technology while using it. Experience also reduces technological anxiety and strengthens the user’s computer self-efficacy and thus increases the perceived enjoyment and strengthens suitability for occupational requirements. Additionally, according to the UTAUT2 model, experience is a mediating variable. Accumulation of experience through repeated use of technology leads to the development of habit, which is one of the strongest predictors of continued use of technology. Moreover, experience increases effort expectancy, because the technology is perceived as simpler to operate. This can be described as follows: Accumulation of experience → higher technological confidence → high enjoyment → more behavioral automation → high positive attitudes and more effective learning. Therefore, the second hypothesis is as follows:

*H2*: Greater experience of students with GenAI tools will be positively associated with higher perceived learning effectiveness and more positive attitudes toward these tools.

### 3.3 Ethical knowledge in using GenAI tools

Ethical knowledge is not just normative knowledge but a cognitive mechanism that reduces perceptual ambiguity. According to the TAM3, when a user understands the limits of legitimate use of technology, the feeling of uncertainty and fear of misuse are reduced, a situation that directly corresponds to computer anxiety, which is a factor known to harm the perception of ease of use. Reducing technological anxiety improves PEOU, which in turn increases PU. Ethical knowledge also acts as a social-cognitive resource that allows for safer and more professional interactions with technology in a way that complies with social requirements, a situation that strengthens the user’s Image and their Subjective Norm. Within the framework of the UTAUT2 model, ethical knowledge can be seen as a conceptual extension of facilitating conditions, not only as a technical infrastructure but also as a knowledge-based social-regulatory infrastructure that enables responsible and informed use of technology. Moreover, when the user feels that they are operating within a clear normative framework, performance expectancy is strengthened because technology is perceived as a tool that can be legitimately and effectively integrated into learning processes. This can be described as follows: Ethical knowledge → higher technological confidence → strengthen the image and norms compliance → higher technological performance → high positive attitudes and more effective learning. Therefore, the third hypothesis is as follows:

*H3*: Higher levels of ethical knowledge of students regarding the responsible use of GenAI tools will be positively associated with perceived learning effectiveness and favorable attitudes toward these tools.

### 3.4 Merits and drawbacks of GenAI tools

In the TAM3 framework, the user’s ability to identify advantages alongside disadvantages of GenAI reflects deep analytical cognitive processing and advanced understanding of the technology, a condition that strengthens the PU. In addition, a user who critically evaluates the technology tends to develop Result Demonstrability, a tangible understanding of the technology’s contribution to performance. This understanding strengthens the perception of usefulness because the value derived from using the technology is not perceived as an abstract promise but as an observed actual result. Awareness of the technology’s limitations may also improve objective usability, as a user learns to adapt usage patterns to the technology’s capabilities and avoid ineffective use. According to the UTAUT2 model, a critical assessment of advantages and disadvantages reflects a cognitive process similar to price value assessment, even when the price is not necessarily expressed in direct monetary terms but cognitive ones, such as the investment of mental resources to learn the technology. When the perceived benefit exceeds the perceived cost, the feasibility is strengthened, and the adoption of the technology increases. This can be described as follows: Understanding the pros and cons → developing critical and objective evaluation based on results → higher technological expectations → high positive attitudes and more effective learning. Therefore, the fourth hypothesis is as follows:

*H4*: Students who demonstrate a balanced merits and drawbacks perception using GenAI tools will report higher perceived learning effectiveness and more positive attitudes toward these tools.

### 3.5 Technological orientation, gender, and age

The TAM3 provides a micro-psychological framework for differences between users using the Anchor variables. Students with a high technological orientation are expected to demonstrate greater computer self-efficacy in their ability to use GenAI, which increases the perception of ease of use and PU. In addition, the factor computer anxiety indicates that levels of technological anxiety may differ between gender, age groups, and students with different levels of technological orientation, thereby influencing learning efficiency and attitudes toward technology. Furthermore, computer playfulness, which describes a tendency to explore technologies spontaneously, is more common among users with a high technological affinity, which increases perceived enjoyment and actual use. In addition, with the accumulation of experience, adjustments occur, leading to more accurate perceptions of the usefulness of technology, a process that may explain differences in the perception of learning effectiveness and attitudes. The UTAUT2 model incorporates moderator variables such as age, gender, and experience, which influence the strength of the relationships between performance expectations, effort expectations, and intention to use technology. Age may affect effort expectancy, as younger users tend to perceive new technologies as simpler to use, while older users may attribute higher weight to perceived effort. These differences may be reflected in different attitudes and even in the educational benefit derived from technology. Gender affects the way men and women evaluate PU versus ease of use. Likewise, technological orientation, which is conceptually close to experience, may strengthen performance expectancy and the development of habit, thus leading to more effective use and improved learning outcomes. This can be described as follows: Differences in personal characteristics → literacy development and technological education → rapid technological adaptation → improvement in technological capabilities and high computer playfulness → more experience and automated habit → high performance expectancy and perceived enjoyment → high positive attitudes and more effective learning. Therefore, the five and six hypotheses are as follows:

*H5*: A higher level of technological orientation of students will be positively associated with perceived learning effectiveness and positive attitudes toward the use of GenAI tools.

*H6*: Significant differences are expected to be found across gender and age groups of students in learning effectiveness and attitudes toward using GenAI tools.

Figure 1 illustrates the integration of the two theoretical approaches, incorporating the dependent and independent variables of the six tested research hypotheses.

**Figure 1:**
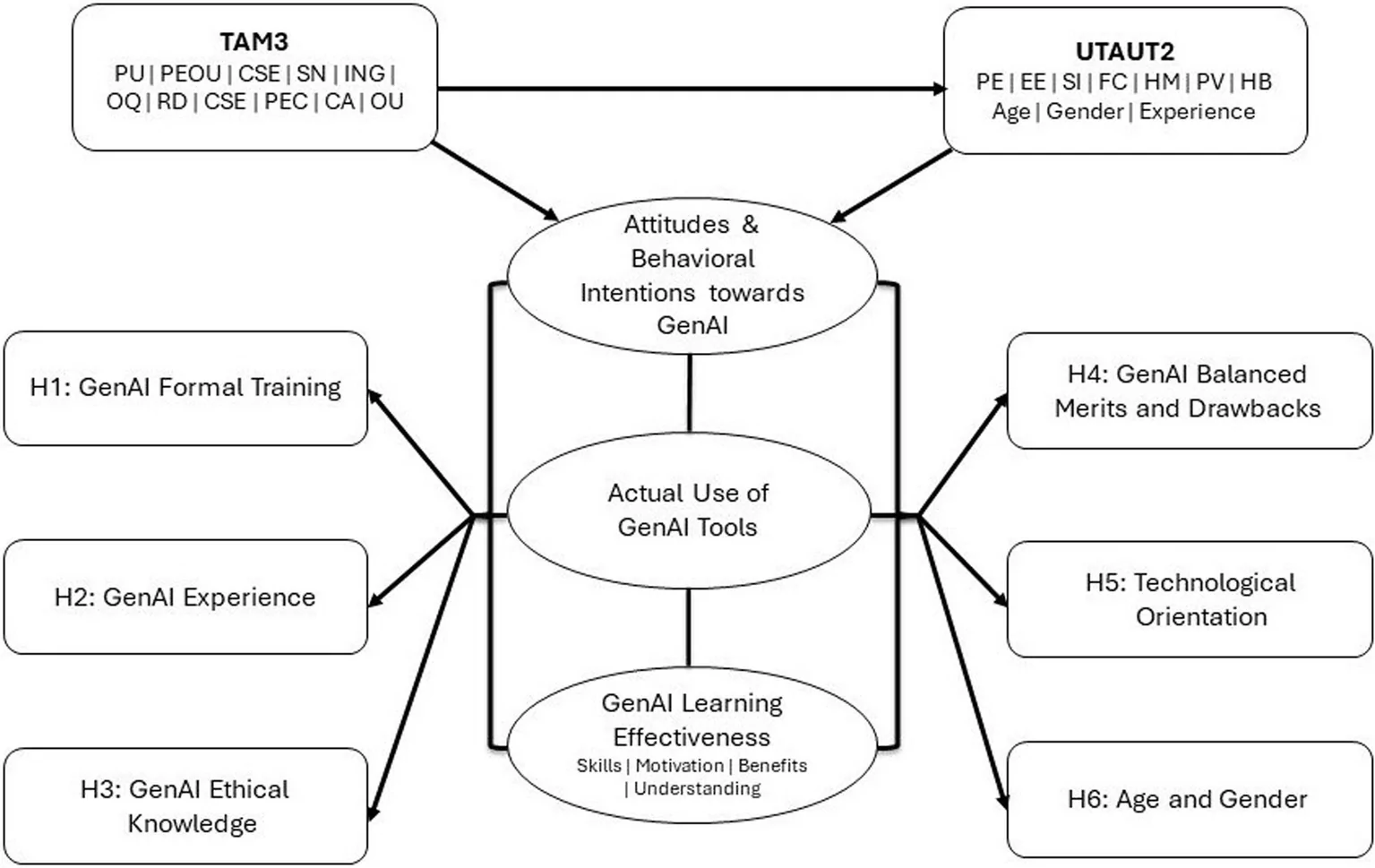
Integrated TAM3–UTAUT2 framework predicting attitudes and learning effectiveness using GenAI tools.

## 4 Methods

### 4.1 Sample population

A total of 566 students from Israel’s third-largest university (out of eleven) participated in the current study. Of them, 425 students (75%) identified as female and 141 (25%) as male. The participants’ ages ranged from 20 to 52 years (*N* = 566), with a mean age of 23.17 years (SD = 6.64). The students who participated in the sample studied in two faculties: Engineering (36%) and Social Sciences (64%). Regarding their academic status, most of the participants were bachelor’s degree students (78.5%), while 18.7% were enrolled in master’s programs, and 2.8% were PhD candidates. In terms of technological orientation within their academic field, most students (69.5%) reported being engaged in a technologically oriented degree program, whereas 30.5% were not.

### 4.2 Data collection method

The vast majority of studies cited in the literature review have successfully utilized a quantitative approach employing a multiple-choice questionnaire to examine students’ attitudes and perceptions toward GenAI technologies. Accordingly, in this study, quantitative methodology was selected as the most appropriate approach for data collection. The data were acquired via an online, self-report instrument created with Google Forms, which was administered to university students during the first semester of 2026. All participants were informed of the anonymity of their submissions and the sole use of the data for research, leading the research team to presume a high degree of veracity in the self-reported information. The online questionnaire was divided to four sections that designed to examine the following: (1) usage academic patterns, ethical knowledge, and experience of students with GenAI tools, (2) their attitudes toward the GenAI-supported learning process and their satisfaction with these tools, (3) their perceptions of the effectiveness of GenAI technologies in learning, and (4) the necessary demographic data for the sample.

The questionnaire employed in this study constitutes a contextually adapted iteration of the measures developed and examined by Carmi (2025). Recognizing the necessity of aligning the instrument precisely with specific research constructs, a comprehensive quality assurance process was undertaken. The psychometric soundness of the resultant questionnaire was established through content validity and face validity procedures. Initially, a research associate with domain expertise conducted a thorough evaluation to verify the representativeness of all items relative to the study’s variables. This expert review prompted the revision of several statements in the questionnaire. The refined instrument was subsequently deployed in an exploratory pilot test among a randomized cohort of students who participated in the first study on this subject (N = 42). Participant feedback from the pilot phase facilitated the final refinement of the items’ wording for research goals.

## 5 Data analysis results

Analysis of students’ responses in the study showed that using the GenAI tool in academic learning, ChatGPT was by far the most frequently used platform (58.6%), followed by Gemini (20.7%), Claude (10.1%), and other tools such as Copilot, Perplexity, and DALL·E 2 (12.4%). Only a small minority of the students (3.5%) reported using no GenAI tools at all. Concerning the need for institutional ethical guidelines for students on the use of GenAI, 41.5% of students supported the formulation of such guidelines, 30.1% were opposed, and 28.4% reported being unsure. When asked about the perceived merits of using GenAI in academic settings, students most noted time and effort savings (38.9%), improved performance and quality of work (30.9%), access to advanced technological tools (28.3%), and increased efficiency and productivity (27.4%). Additional benefits included enhanced learning and research opportunities (24.7%) and better preparation for a technology-driven job market (23.9%). However, notable perceived drawbacks reported by participants included overreliance on GenAI tools (51.2%), impaired critical thinking and creativity (43.3%), and diminished academic skills (33.6%). Concerns about privacy and data security (12.4%), copyright and intellectual property issues (10.6%), and the risk of misinformation or low reliability (4.4%) were also cited. As the participants were allowed to select multiple responses, the total percentages for GenAI tools used, and perceived merits and drawbacks exceeded 100% (Table 1).

**Table 1:** Types of tool use and perceived merits and drawbacks of GenAI usage (*N* = 566).

Variable	Category	%
Use of GenAI tools	ChatGPT	58.6%
	Gemini	20.7%
	Claude	10.1%
	Other tools (e.g., Copilot, Perplexity, DALL·E2)	12.4%
	No use of GenAI tools	3.5%
Perceived merits of GenAI tools	Time and effort saving	38.9%
	Improved performance and work quality	30.9%
	Access to innovative and technological tools	28.3%
	Increased efficiency and productivity	27.4%
	Expanded learning and research opportunities	24.7%
	Better preparation for a technology-driven job market	23.9%
Perceived drawbacks of GenAI tools	Overdependence on GenAI tools	51.2%
	Impaired critical thinking and creativity	43.3%
	Impaired academic skills	33.6%
	Privacy and data protection concerns	12.4%
	Copyright and intellectual property issues	10.6%
	Risk of misinformation or low reliability	4.4%

Among the total items in the GenAI questionnaire, 48 items were rated on a 6-point Likert scale, ranging from 1 (“not at all”) to 6 (“very much”). The questionnaire included four subscales: (1) GenAI training (7 items; *α* = 0.72), (2) GenAI ethical knowledge (6 items; *α* = 0.69), (3) attitudes toward GenAI tools (15 items; *α* = 0.95), and (4) learning effectiveness (20 items) divided to skills (5 items; *α* = 0.91), motivation (5 items; *α* = 0.86), benefits (5 items; *α* = 0.91), and understanding (5 items; *α* = 0.85). Internal consistency for all subscales ranged from acceptable to excellent, as indicated by Cronbach’s alpha coefficients (see Appendix A).

Given the cross-sectional design and the use of self-reported measures collected through a single questionnaire, the study assessed the potential presence of common method bias (CMB). Harman’s single-factor test was conducted by entering all measurement items into an exploratory factor analysis without rotation. The first unrotated factor accounted for 39.3% of the total variance, which is below the commonly used 50% threshold. This result indicates that no single factor accounted for the majority of the covariance among the measurement items, suggesting that common method bias is unlikely to represent a substantial threat to the findings, thereby supporting the suitability of the data for further structural hypothesis testing.

All statistical analyses were conducted using IBM SPSS Statistics version 28 and R version 4.4.0 (R Core Team, 2024). Structural equation modeling (SEM) and confirmatory factor analysis (CFA) were performed using the lavaan package version 0.6–17 (Rosseel, 2012) in R. The statistical analysis plan included four sequential stages. First, descriptive statistics (means, standard deviations, and frequency distributions) were computed for all study variables. Bivariate associations among the main variables, such as attitudes toward GenAI, perceived learning effectiveness, GenAI tools training, and ethical knowledge regarding GenAI, were examined using Pearson correlation coefficients. Second, a CFA was conducted to validate the measurement models for two latent constructs: GenAI experience and perceived GenAI learning effectiveness. Model fit was evaluated using the Comparative Fit Index (CFI), Tucker–Lewis Index (TLI), Root Mean Square Error of Approximation (RMSEA), and Standardized Root Mean Square Residual (SRMR), with established cutoff criteria used to determine acceptable model fit (Hu and Bentler, 1999).

Third, a SEM was estimated to test the hypothesized relationships between the independent variables GenAI tools training, ethical knowledge regarding GenAI, perceived merits and drawbacks of GenAI, technological orientation, and the latent variable of GenAI experience and the two main outcome variables: attitudes toward GenAI and perceived GenAI learning effectiveness (also modeled as a latent construct). Finally, to examine gender differences in the main study variables, a multivariate analysis of variance (MANOVA) was performed with gender as the independent variable and the GenAI -related variables as dependent variables. Where a significant multivariate effect was detected, follow-up univariate tests were conducted to assess specific group differences. All statistical tests were two-tailed, and the significance level was set at α = 0.05 and α = 0.01.

To examine the associations between students’ learning effectiveness, attitudes, GenAI training, and ethical knowledge in using GenAI for academic purposes, a Pearson correlation analysis was conducted. As shown in Table 2, GenAI training was positively associated students’ attitudes (*r* = 0.25, *p* < 0.001) and with all four dimensions of learning effectiveness as follows: skills (*r* = 0.23, *p* < 0.001), motivation (*r* = 0.24, *p* < 0.001), benefits (*r* = 0.21, *p* < 0.001), understanding (*r* = 0.21, *p* < 0.001). Similarly, ethical knowledge regarding GenAI showed significant, albeit lower, positive correlations with students’ attitudes (*r* = 0.16, *p* < 0.001) and learning effectiveness variables as follows: skills (*r* = 0.19, *p* < 0.001), motivation (*r* = 0.18, *p* < 0.001), benefits (*r* = 0.19, *p* < 0.001), understanding (*r* = 0.20, *p* < 0.001). These results suggest that students with more extensive training and greater ethical knowledge have favorable attitudes toward GenAI and tend to perceive higher levels of academic learning effectiveness in using GenAI tools.

**Table 2:** Pearson correlations between students’ attitudes, learning effectiveness, GenAI training, and ethical knowledge (*N* = 566).

	Attitudes	Learning effectiveness			
		Skills	Motivation	Benefits	Understanding
Students’ GenAI training	0.25***	0.23***	0.24***	0.21***	0.21***
Students’ GenAI ethical knowledge	0.16***	0.19***	0.18***	0.19***	0.20***

***p < 0.001.

The presence of significant correlations among all core study variables led the researchers to hypothesize that a comprehensive structural model would best capture the underlying patterns in the data. Accordingly, a SEM analysis was conducted using the lavaan package in R statistical programming language (Rosseel, 2012). In this model, the independent variables included prior institutional training on GenAI tools, ethical knowledge in using GenAI, perceived merits and drawbacks of GenAI (both modeled as continuous variables), and technological orientation of the academic program (coded as binary: technological vs. non-technological). In addition, students’ experience with GenAI tools was modeled as a latent variable derived from three examined indicators: the number of different GenAI tools used by students, the frequency of GenAI use, and the extent of GenAI use in academic settings. The dependent variables in the model comprised students’ attitudes toward GenAI, and a latent construct of learning effectiveness with GenAI tools, which was defined by four examined indicators: skills, motivation, benefits, and understanding.

Prior to estimating the full SEM, a preliminary CFA was conducted to validate the measurement models of the two latent constructs: GenAI students’ experience and perceived GenAI learning effectiveness. The CFA results indicated a good fit of the measurement model to the observed data, based on commonly accepted thresholds (Hu and Bentler, 1999). Specifically, the model demonstrated strong fit across the principal indices, with CFI = 0.971, TLI = 0.952, RMSEA = 0.042, and SRMR = 0.035. These values suggest that the latent constructs were adequately represented by their observed indicators and supported the continuation to the full SEM analysis. The reliability and validity of the latent constructs were further assessed using the standardized factor loadings and the relevant reliability and validity criteria. As presented in Figure 2 and detailed in Table 3, the structural model explained 54.5% of the variance in students’ attitudes toward GenAI and 48.1% of the variance in learning effectiveness through GenAI technologies. These R² values indicate that the structural model accounted for substantial proportions of variance in both endogenous outcome constructs.

**Figure 2:**
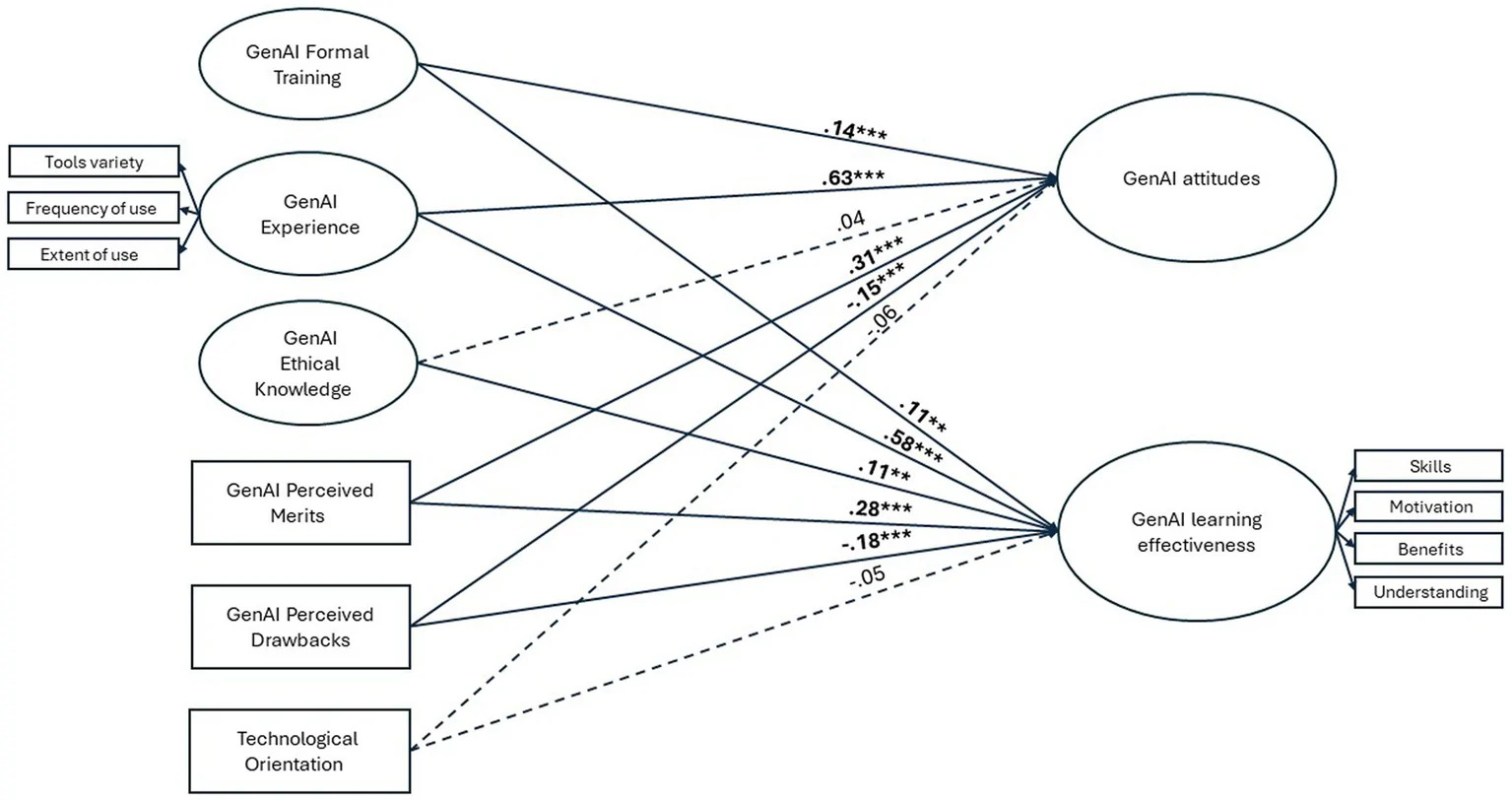
SEM predicting students’ attitudes and learning effectiveness using GenAI. **p < 0.01, ***p < 0.001.

**Table 3:** Standardized regression coefficients, *p*-values, and effect sizes for predictors of students’ attitudes and learning effectiveness via GenAI.

Effect	*β*	*p*-value	Effect size	*R* ^2^
GenAI attitudes				0.545
Training → attitudes	0.14***	<0.001	Small	
Experience → attitudes	0.63***	<0.001	Large	
Ethical knowledge → attitudes	0.04	0.248	—	
Merits → attitudes	0.31***	<0.001	Medium	
Drawbacks → Attitudes	−0.15***	<0.001	Small	
Tech orientation → attitudes	−0.06	0.056	—	
GenAI learning effectiveness				0.481
Training → effectiveness	0.11**	0.004	Small	
Experience → effectiveness	0.58***	<0.001	Large	
Ethical knowledge → effectiveness	0.11**	0.003	Small	
Merits → effectiveness	0.28***	<0.001	Medium	
Drawbacks → effectiveness	−0.18***	<0.001	Small	
Tech orientation → effectiveness	−0.05	0.147	—	

**p < 0.01, ***p < 0.001.

Among the predictors of GenAI attitudes, students’ experience via GenAI emerged as the strongest and most influential factor (*β* = 0.63, *p* < 0.001), with a high effect size, suggesting that students with greater exposure to GenAI tools and platforms report significantly more favorable attitudes toward using GenAI tools. Students’ training on GenAI was also a significant predictor (*β* = 0.14, *p* < 0.001), though with a low effect size, indicating that formal instructional to GenAI technologies contributes very little to students’ attitudes. This finding may be because of the lack of comprehensive and regulated institutional training. Perceived merits of GenAI showed a medium-sized positive effect on attitudes (*β* = 0.31, *p* < 0.001), whereas perceived drawbacks of GenAI had a significant low negative effect (*β* = −0.15, *p* < 0.001), indicating that students’ ambivalence or concerns regarding GenAI diminish their positive attitudes. Ethical knowledge regarding the GenAI use (*β* = 0.04, *p* = 0.248) and the technological orientation of students (*β* = −0.06, *p* = 0.056) did not significantly predict attitudes in this model.

Regarding GenAI learning effectiveness, GenAI experience also emerged as the strongest predictor (*β* = 0.58, *p* < 0.001), with a high effect size, finding that reinforces the centrality of direct interaction with GenAI tools in shaping students’ assessments of their effectiveness in academic learning. Additionally, perceived merits of GenAI demonstrated a medium positive effect (*β* = 0.28, *p* < 0.001), and students’ training on GenAI tools (*β* = 0.11, *p* = 0.004) and ethical knowledge regarding GenAI use (*β* = 0.11, *p* = 0.003) both had low but significant effects on perceived learning effectiveness. Perceived drawbacks regarding GenAI tools were again significant (*β* = −0.18, *p* < 0.001) with a low negative effect, suggesting that concerns about GenAI can erode students’ perceptions of their usefulness. Technological orientation was not a significant predictor of perceived effectiveness (*β* = −0.05, *p* = 0.147).

To examine gender differences across the main study variables, a multivariate analysis of variance (MANOVA) was conducted with gender as the independent variable and all primary research variables as dependent variables. As presented in Table 4, the analysis revealed a statistically significant multivariate effect of gender on the combined dependent variables, indicating meaningful group differences across several domains. Univariate follow-up tests indicated that women reported significantly higher levels of GenAI training (*M* = 2.99, SD = 0.91) compared to men (*M* = 2.75, SD = 0.90), *F*(1, 479) = 6.85, *p* = 0.009. However, men reported significantly higher GenAI experience (*M* = 4.39, SD = 1.33) than women (*M* = 4.09, SD = 1.25), *F*(1, 479) = 5.34, *p* = 0.021. In addition, men demonstrated significantly higher ethical knowledge (M = 4.15, SD = 0.97) than women (M = 3.83, SD = 1.00), *F*(1, 479) = 9.40, *p* = 0.002, suggesting a gender gap in ethical awareness in favor of the former.

**Table 4:** Gender differences in students in training, experience, ethical knowledge, attitudes, and learning effectiveness regarding GenAI.

Dependent variables	Men (*N* = 141)		Women (*N* = 425)		*F*-value	*p*-value
	*M*	*SD*	*M*	*SD*		
Training	2.75	0.90	2.99	0.91	6.85**	0.009
Experience	4.39	1.33	4.09	1.25	5.34*	0.021
Ethical Knowledge	4.15	0.97	3.83	1.00	9.40**	0.002
Attitudes	4.75	1.10	4.52	1.05	4.45*	0.035
Effectiveness - Skills	4.01	1.29	3.70	1.34	5.11*	0.024
Effectiveness - Motivation	3.78	1.34	3.59	1.25	2.08	0.149
Effectiveness - Benefits	4.51	1.37	4.25	1.27	3.69*	0.045
Effectiveness - Understanding	4.60	1.12	4.37	1.17	3.57*	0.050

*p < 0.05, **p < 0.01.

Significant gender differences also emerged in the outcome variables. Men exhibited more positive attitudes toward GenAI (*M* = 4.75, SD = 1.10) compared to women (*M* = 4.52, SD = 1.05), *F*(1, 479) = 4.45, *p* = 0.035. In terms of GenAI learning effectiveness, men scored higher than on skills (*M* = 4.01 vs. 3.70), *F*(1, 479) = 5.11, *p* = 0.024, benefits (*M* = 4.51 vs. 4.25), *F*(1, 479) = 3.69, *p* = 0.045, and understanding (*M* = 4.60 vs. 4.37), *F*(1, 479) = 3.57, *p* = 0.050. However, no significant gender difference was found for motivation, *F*(1, 479) = 2.08, *p* = 0.149. Taken together, the findings suggest that while women students report greater engagement in formal GenAI training, their male counterparts exhibit higher levels of hands-on experience, stronger attitudes toward GenAI, and greater perceived learning effectiveness across multiple domains. Notably, men also demonstrated significantly higher ethical knowledge, pointing to a potential gap in the integration of ethical instruction within training contexts targeted at women learners (Figure 3).

**Figure 3:**
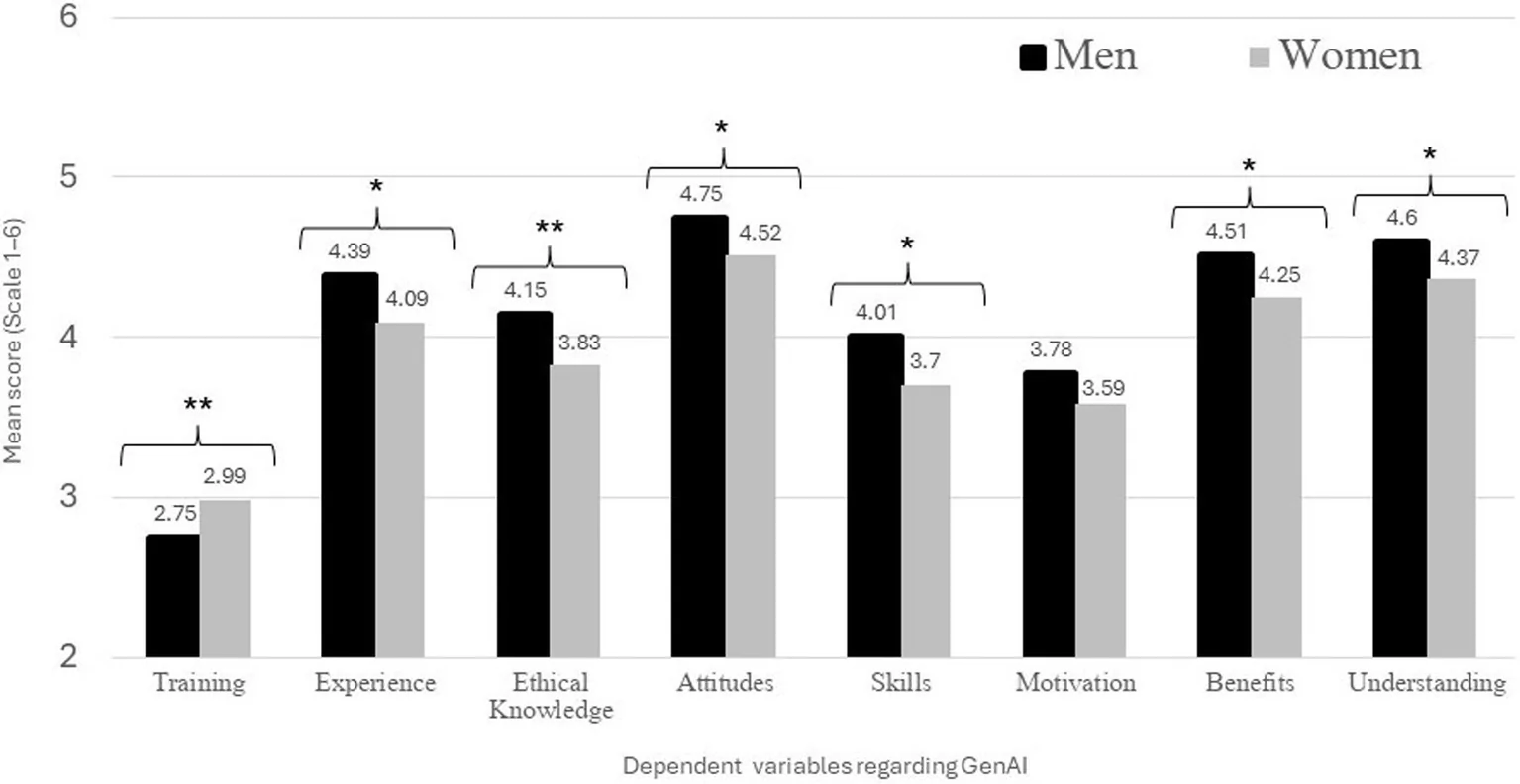
Gender differences in students in training, experience, ethical knowledge, attitudes, and learning effectiveness regarding GenAI tools. *p < 0.05, **p<0.01.

## 6 Discussion

The study aimed to examine the relationships between students’ training on GenAI tools, their experience using GenAI, ethical knowledge regarding the use of these tools, perceived merits and drawbacks toward GenAI, the influence of technological orientation, gender and age on the adoption of these technologies, and attitudes toward GenAI tools and the effectiveness of academic learning by using them. The core findings illuminate the centrality of direct students’ experience with GenAI, the significance of providing training on these tools, the mixed opinions regarding ethical guidelines, the gender differences using tools, and the gap between perceived benefits and primary concerns arising from using these intelligent technologies. From a theoretical perspective, these relationships align with the technology adoption frameworks of the TAM3 and the UTAUT2, which emphasize that perceptions of usefulness, effort, and contextual experience shape both attitudes and behavioral outcomes.

The regression analyses consistently and unequivocally show that students’ experience using GenAI is the strongest and most influential predictor for both favorable attitudes toward the tools (β = 0.63) and high perceived learning effectiveness (*β* = 0.58). The strength of this relationship suggests that technological adoption and the assessment of utility are primarily driven by experiential learning and the pragmatic alignment of the tools to individual academic needs. Moreover, these findings underscore that continuous, direct interaction most significantly shapes students’ evaluation of the tools and their perception of the tools’ contribution to their learning. Gaining experience of students interpreted as a process of uncertainty reduction and the practical adaptation of the tool to specific academic needs, leading to more positive adoptions. This finding is consistent with empirical studies that identified students’ hands-on experience with GenAI systems as a key determinant of technology acceptance and perceived academic benefits (Dwivedi et al., 2023; Kasneci et al., 2023). These studies show that frequent interaction with GenAI tools enhances perceptions of usefulness and learning support, which aligns with the mechanisms proposed by the theoretical models. Similarly, recent studies on GenAI adoption in higher education demonstrate that direct interactions with GenAI tools significantly predict both positive attitudes and academic engagement (Carmi, 2025; Daher and Hussein, 2024; Duy et al., 2025).

While formal GenAI training was found to be positively related to both attitudes and learning effectiveness, its predictive power in the regression analysis was relatively weak (*β* = 0.14 for attitudes and *β* = 0.11 for effectiveness). Ethical knowledge showed an even lower correlation, with no significant prediction of attitudes (*β* = 0.04) and only marginal prediction of learning effectiveness (*β* = 0.11). These results may be attributed not only to the limited reliability of the ethical knowledge measure (α = 0.69), which may have attenuated the observed relationship, but also to a lack of consistency or comprehensiveness in current institutional instruction, as found in previous studies (Carmi, 2025; Gasaymeh et al., 2024; Elchaghaby and Wahby, 2025). These outcomes are also consistent with studies indicating that training alone does not necessarily translate into sustained technology adoption unless it is complemented by authentic learning activities and practical usage (Chan and Hu, 2023; Crompton and Burke, 2023). From a theoretical standpoint, the TAM3 assumes that training on technological tools strengthen PEOU and self-efficacy of students, yet the current findings suggest that experiential interactions, which lead to cumulative experience, may play a more dominant role than formal instruction in shaping students’ attitudes toward GenAI tools and their perception of learning effectiveness from using these tools.

Regarding ethical knowledge, the results suggest a psychological separation between the theoretical understanding of ethical rules and the perception of the tool’s practical value. Students differentiate between understanding the rules and the experience and effectiveness of the tools themselves; theoretical knowledge about ethics does not necessarily translate into a more favorable general attitude toward the tools or improving learning effectiveness by using them. This gap may indicate a relative failure of formal institutional intervention compared to the formative power of practical interaction through personal experience purchased by the student. Similar to these, the Bećirović et al. (2025) findings suggest that perceived academic advantages, instead of ethical or normative concerns, serve as the primary determinants of student attitudes toward GenAI, as ethical factors did not significantly influence perceptions of the technology’s utility or usability. Cotton et al. (2024) also mention that although students recognize ethical concerns regarding GenAI in higher education, these concerns do not always translate into changes in attitudes toward using the technology for academic tasks. These findings are consistent with that of Floridi (2023) who argues that ethical awareness of GenAI focuses on principles such as accountability, transparency, and fairness and not necessarily influence PU or technology acceptance.

In addition, the finding that 41.5% of students support the formulation of ethical guidelines indicates a clear demand for institutional frameworks governing the responsible use of GenAI tools. A significant portion of students are not rejecting the tools but are seeking a defined regulatory framework to help navigate the academic and ethical benefits and risks, such as overreliance or concerns over privacy. This support for ethical guidelines aligns with the desire to enhance learning effectiveness and mitigate concerns about weakening academic skills. This result is consistent with those of recent studies examining GenAI governance in higher education, which report increasing student demand for formal institutional policies and clear ethical guidelines to regulate the use of GenAI tools in academic contexts (Carmi, 2025; Cotton et al., 2024; Dwivedi et al., 2023). Within the theoretical framework of UTAUT2, this demand can be interpreted as evidence of the importance of facilitating conditions, suggesting that institutional structures and clear usage policies play an important role in supporting responsible technology adoption.

Furthermore, student responses in the study reveal significant ambivalence regarding the use of GenAI tools in academic studies. On the one hand, students perceive clear merits in productivity and efficiency, particularly time and effort savings (38.9%), improved quality of academic work (30.9%), and increased efficiency and productivity (27.4%). These perceived benefits were strong positive predictors of both favorable attitudes (*β* = 0.31) and perceived learning effectiveness (*β* = 0.28). On the other hand, students expressed a dominant and widespread concern regarding the potential negative effects of GenAI use in academic learning. Over half of them expressed concern about overreliance on these tools (51.2%) and significantly feared impaired critical thinking and creativity (43.3%) and diminished academic skills (33.6%). These concerns were found to be a significant decline (though low-impact) in both favorable attitudes (*β* = −0.15) and perceived learning effectiveness (*β* = −0.18). The findings show that students are internally negotiating between maximizing short-term academic performance and preserving long-term cognitive development, highlighting a crucial need for academic institutions’ pedagogical interventions. This ambivalence also suggests that students are well aware of the destructive potential of GenAI technologies alongside their productive potential, indicating a degree of maturity in perceiving risks and benefits inherent in using these tools.

These findings align with those of prior empirical studies applying the TAM and UTAUT frameworks, which demonstrate that PU and performance expectancy represent the most powerful predictors of technology acceptance (BinJwair, 2025; Dwivedi et al., 2023; Farrokhnia et al., 2023; Lim et al., 2023). The current results reinforce the central role of perceived benefits and proven performance in shaping perceptions of effectiveness and attitudes toward GenAI-based learning technologies. Furthermore, this dual perception of students has also been identified in recent studies on students’ perceptions regarding the integration of GenAI tools. Cotton et al. (2024) have found that students simultaneously acknowledge the efficiency advantages of GenAI while expressing concerns about the erosion of independent thinking skills. Similar results were also found in Almassaad et al. (2024), Chan and Hu (2023), Daher and Hussein (2024), and Sami et al. (2025).

Contrary to our belief, the technological orientation of the students in the sample, examined by belonging to a technological/non-technological curriculum, was not found to be a significant predictor of attitudes and learning effectiveness. The reason for this may very well be that GenAI tools are widely available online; there is a high awareness of their power and capabilities, they have widespread generic utility, and they currently cross scientific disciplines. This could be reinforced by the finding that only a slight percentage (3.5%) of all students reported not using GenAI tools at all for their studies. Furthermore, while technological orientation plays a central role as an explanatory mechanism underlying individual differences in the adoption and use of GenAI tools, the results of this study highlight that university students can adopt the use of GenAI tools and effectively leverage them to improve learning outcomes across a range of knowledge domains, including skill development, perceived benefits, and conceptual understanding, regardless of the technological orientation of the academic path.

These results contrast with research findings that have shown that students enrolled in technological fields consistently demonstrate higher levels of digital competence, self-efficacy, and performance expectancy, which in turn enhance their behavioral intention to adopt GenAI tools and their attitudes toward the technologies (BinJwair, 2025). Within the UTAUT framework, these differences were reflected in stronger performance expectancy and effort expectancy among students with technological backgrounds, whereas students from non-technological disciplines tend to rely more heavily on facilitating conditions and social influence to support adoption GenAI (Tran and Le, 2025). Similarly, from the TAM perspective, students in technology-oriented programs reported higher PU and PEOU, primarily because of their prior experience and greater technological confidence (Liu et al., 2025), while students from non-technological fields often showed lower perceived competence and higher levels of uncertainty when using GenAI tools (Prohorovs et al., 2026).

In addition, regarding the analysis of demographic variables, no statistically significant effect was found for age. This lack of significance is likely attributable to the age homogeneity of the sample, as the mean age (*M* = 23) was highly skewed toward the lower bound of the student population range. However, the results of the gender analysis revealed a complex and interesting picture regarding the use of GenAI technologies. Overall a statistically significant multivariate effect of gender was found, indicating gender differences in the perceptions, attitudes, and use of these technologies. The key results concern the gender inverse relationship between training and experience, as well as gender gaps in ethical knowledge, attitudes, and the perceived learning effectiveness of GenAI. The most salient finding is the difference between training and actual experience. Women reported higher levels of GenAI training, suggesting a more active engagement in formal courses or workshops for using these technologies, while men reported significantly higher actual experience with GenAI, as reflected in their higher mean score. This finding raises the possibility that women need more formal guidance for using GenAI tools, while men acquire more experience autonomously through self-exploration and independent use of the technologies, even outside of formal learning settings.

Another gender difference concerns ethical knowledge in using GenAI. Although women reported higher levels of formal training than men, the latter reported significantly higher ethical knowledge than the former. The area of ethics in using AI tools may not be sufficiently emphasized and comprehensive in student-oriented training contexts. This gender gap in ethical knowledge has significant implications, given that the responsible and ethical use of GenAI is a central issue in academic and professional discourse. In another aspect, men exhibited significantly more positive attitudes toward using GenAI tools. Moreover, they rated their learning effectiveness using GenAI higher than women on three variables: skills, benefits, and understanding. More positive attitudes combined with practical experience with the tools may create more effective learning, influence their assessment of the learning benefits, and encourage them to derive greater benefit from these tools. While no gender differences were found in the motivation variable, the higher scores of men on the other measures of learning effectiveness indicates that despite similar levels of motivation, the experience of use and belief in the learning abilities with GenAI differ significantly by gender. As the use of these technologies among students in academic frameworks increases, it is significant that gender gaps will be closed.

Finally, this finding is consistent with the moderating role of mediating variables proposed in the UTAUT model, suggesting that gender and experience may influence both technology adoption and performance outcomes in using it. Empirical studies based on UTAUT that have focused on higher education students provide mixed but converging evidence regarding gender differences in GenAI adoption and use. Kinskofer and Tulis (2025) found in their study on students, that men demonstrated higher levels of behavioral intention, PU, and actual use of GenAI tools, while their women counterparts reported lower perceived competence, less motivation, and higher levels of perceived risk. Similarly, Elnaem et al. (2025) found higher levels of behavioral intention to adopt GenAI, performance expectancy, and effort among men compared to women, and Maxwell et al. (2025) reported that men compared to women expressed more positive attitudes and used GenAI tools more. However, a meta-analysis study suggests that gender differences may not significantly influence actual usage behavior but operate at the level of attitudes or perceptions (Diao et al., 2024), while no significant influence of gender on the adoption or use of GenAI tools were found in others (Carmi, 2025; Prohorovs et al., 2026).

## 7 Conclusions and recommendations

Several conclusions emerge from the research findings. The main conclusion of the study is that students’ cumulative personal experience in learning with GenAI tools is the cognitive-behavioral factor that most determines learning effectiveness and positive attitudes reported, with positive aspects strengthening the perception toward GenAI and negative aspects weakening it. Academically, this indicates that theoretical knowledge alone about GenAI technologies is not sufficient; perceived learning effectiveness and positive attitudes are built through learning by doing. Both are not only related to technical ability but also likely reflect students’ ability to integrate GenAI in a meaningful and productive way into their thinking and learning processes. Therefore, direct interaction and accumulating experiences are critical to shaping students’ assessment of GenAI’s usefulness for their academic study.

Another conclusion finds that students must undergo comprehensive educational training on GenAI tools used for academic learning purposes. Specific training in using GenAI tools not only improves technical skills and understanding of the benefits but also strengthens students’ motivation to learn and their positive attitudes toward the use of tools and allows them to frame tools of GenAI as a legitimate and beneficial academic for learning. Moreover, focused training, according to the needs of each disciplinary field, provides students with mental models and effective work processes that make it easier for them to identify and recognize the usefulness of the tools. Training helps transform unstructured experience into a more structured and effective experience.

Additionally, increasing students’ awareness and ethical knowledge in formal training settings is not just a matter of compliance with rules but directly contributes to building trust and a better understanding of the correct and productive way to integrate GenAI tools into their studies. Psychologically, students who express concerns create a mental barrier for themselves that prevents them from realizing the full potential of the technological tool or acknowledging it. Ethical understanding may reduce concerns and uncertainty, thereby improving students’ perceptions of learning effectiveness and attitudes toward using GenAI. Moreover, when students understand the rules for the proper and ethical use of GenAI tools in their learning processes, they may feel more confident using these tools in a binding academic setting. Ethics, in this case, will transform from a limiting rule to a determinant of PU and effectiveness.

Moreover, unlike highly complex technological tools that require specific knowledge, GenAI tools are adaptive, useful, and relatively simple to operate and learn. Although the tools require a unique cognitive skill, this does not necessarily stem from a general inclination toward technology or belonging to technological professions, as this study has found. Despite the short time since the emergence of this high technology, there is already a fundamental need to develop GenAI literacy among students, which is no longer limited to just computing or information technology literacy as it was until recently. Therefore, there is a need for an instructional focus for students on a specific GenAI orientation instead of a general technological one. Finally, instruction on these tools may be differential between men and women according to need and abilities in order to reduce the gender gap found in the study and to optimally prepare students for the daily use of highly intelligent tools in the future.

At the institutional level, our main conclusion is that academic institutions must shift from the pedagogy of prohibiting the use or providing superficial theoretical instructions on to that of controlled, experiential integration of GenAI tools. Educational interventions must focus on designing tasks that promote the use of GenAI as a strategic tool for cognitive augmentation, specifically to address students’ concerns about diminishing critical thinking and overreliance on these tools. Training must be designed to leverage the powerful formative influence of direct experience while embedding assessment mechanisms that support the judicious use of, rather than replacing, higher-order thinking or knowledge. Another institutional conclusion is the immediate need to establish a clear normative climate for students to correctly use GenAI. Academia must take responsibility for generating clear and actionable ethical guidelines that translate theoretical knowledge into an applied norm; such actions will help students navigate complex issues of intellectual property, plagiarism, and academic ethics. Establishing institutional guidelines is not merely a response to student demand but is an essential condition for preserving academic integrity in a rapidly changing technological landscape, enabling students to maximize the benefits of the tools while minimizing both ethical and cognitive risks.

In addition to the study’s conclusions, our conceptual practical recommendations are that academic institutions should create a comprehensive and dynamic conceptual framework regarding the use of GenAI tools. The practical training will provide students with a clear pedagogical framework regarding when and how to use GenAI correctly and legitimately in academic courses. This framing will reduce students’ technological uncertainty, increase their sense of self-efficacy, and improve their perception of control when it comes to learning with these advanced tools. We strongly recommend that educational interventions move away from a focus on theoretical instructions toward fostering experience-based and project-driven learning, which capitalizes on the strength of direct interaction with diverse GenAI tools. Moreover, institutions must design training to directly address students’ cognitive concerns, emphasizing the use of GenAI as a tool for cognitive augmentation and supporting metacognitive skills, rather than merely as a shortcut for academic task completion. We believe that only a strategic integration of clear institutional guidelines with practical experience-based pedagogy can maximize the benefits of GenAI as study tools while safeguarding the integrity of academic learning.

Further to this, our practical recommendations focus on moving to focused experiential learning that includes mandatory academic GenAI-based assignments with a detailed requirement for documenting the processes of using the tools, such as prompt engineering, iterations, and metacognitive control processes. Moreover, as part of the development of a pedagogy of “cognitive empowerment” the use of GenAI tools should be focused on as an amplifier of thinking and not as a substitute for it. For example, students should be instructed to use GenAI only to create drafts or outlines and then to actively improve, validate, and review the output to develop critical thinking and evaluation skills. Furthermore, it is strongly advisable to combine process evaluation and not just product evaluation. Assessment pedagogical models should be developed that emphasize the student’s unique contribution to developing reliable and high-quality knowledge and examining the entire work process, including the stages of interaction with GenAI, in order to address the lack of discernment of “hallucinations” and concern of “overreliance” on GenAI output results.

Additionally, an institutional “ethical climate” for using GenAI technologies must be established, including the formulation of a uniform and clear ethical policy at the institutional level. Institutional managements should formulate binding ethical guidelines regarding the use of GenAI in assignments, exams, and research as early as possible. These guidelines should cover topics ranging from writing a correct citation of GenAI output to defining boundaries for “permissible assistance” and specific intellectual property rules. Moreover, to make ethical knowledge practical, it is advisable not to be satisfied with theoretical lectures but to incorporate practical scenarios in training those present real ethical dilemmas that have arisen because of the academic use of GenAI. This will help translate theoretical knowledge into actual normative behavior. Finally, we recommend using a wide variety of tools to reduce possible biases and test the validity of results. Given the dominance of ChatGPT and Gemini in student use, institutions should encourage exposure and training on a variety of GenAI tools, such as visualization or research tools, to reduce dependence on a single tool and develop a broader, less algorithmically biased GenAI literacy.

## 8 Contribution and limitations

The present study contributes to the growing body of research on GenAI in higher education by theoretically integrating constructs from TAM3 and UTAUT2 with multidimensional measures of learning effectiveness. Three main theoretical contributions can be identified. First, the study expands technology acceptance research by linking adoption models with concrete educational outcomes. While previous research applying TAM or UTAUT frameworks has primarily focused on behavioral intention or technology usage, the current study connects these frameworks not only to attitudes toward technology but also to four dimensions of perceived learning effectiveness: skills development, motivation, perceived benefits, and conceptual understanding. This integration demonstrates how technology acceptance processes and attitudes toward it translate into perceived academic outcomes. Second, the findings highlight the dominant role of experiential learning in shaping attitudes toward GenAI tools. The strong predictive power of experience suggests that repeated interactions with GenAI systems strengthen perceived usefulness and performance expectancy, reinforcing the mechanisms proposed by both TAM3 and UTAUT2. Simultaneously, the relatively weaker effects of formal training for working with GenAI tools suggest that institutional interventions alone may not be sufficient to influence technology adoption and its use unless they are accompanied by opportunities for authentic hands-on engagement. Third, the study introduces the dimension of perceived merits and drawbacks as an important cognitive evaluation mechanism in GenAI adoption. Students simultaneously recognize the productivity benefits of these high technologies and express concerns regarding cognitive dependency and academic skill erosion. In fact, students’ concerns enrich the existing theoretical frameworks that examine the factors of resistance to technology adoption by defining a new type of resistance, which does not stem from a fear of use or technical difficulty but from concern regarding the erosion of cognitive capital and impairment to personal abilities. This dual evaluation provides a more nuanced understanding of technology acceptance in the context of GenAI and suggests that future extensions of TAM or UTAUT-based frameworks may need to explicitly incorporate perceived cognitive risks alongside perceived merits.

The findings of this study carry several important practical implications for higher education institutions that currently require integrating GenAI tools into teaching and learning processes. First, the dominant role of student experience indicates that universities should prioritize experiential learning opportunities with GenAI tools. Instead of relying solely on theoretical instructions, institutions should design pedagogical activities that encourage active experimentation with AI technologies. Structured assignments that require students to critically evaluate AI-generated outputs may simultaneously enhance learning effectiveness and strengthen responsible usage practices. Second, the relatively limited impact of formal training highlights the need to redesign institutional training programs on GenAI. Effective training should not only focus on technical functionality but also integrate ethical awareness, critical evaluation skills, and academic integrity considerations. Such programs can strengthen the facilitating conditions that support responsible GenAI adoption, consistent with the institutional support mechanisms highlighted in UTAUT2. Third, the strong student support for ethical guidelines suggests that higher education institutions should develop clear policies regarding the responsible use of GenAI in academic contexts. Transparent guidelines can reduce uncertainty among students and instructors while maintaining academic integrity and promoting the constructive use of GenAI technologies. Finally, the gender differences identified in the study suggest that institutions should ensure equitable access to training opportunities and encourage inclusive learning environments that support diverse patterns of technological engagement. Addressing these disparities will be crucial as GenAI tools become increasingly integrated into academic learning environments and will form an integral part of the requirements for lecturers in the courses taught.

Notwithstanding the theoretical and empirical contributions of this study, several limitations must also be acknowledged that point to future research directions. First, the use of self-report measures may be affected by perceptual and social desirability biases, especially in the context of innovative technologies such as GenAI. Future studies should, therefore, incorporate more objective indicators of learning performance as academic achievement or task-based assessments to triangulate findings and strengthen the validity of the conclusions. Second, the cross-sectional design constrains the ability to draw causal inferences and to capture the temporal dynamics underlying technology adoption processes. Given that advanced acceptance frameworks emphasize the evolving nature of user beliefs and experiences, future longitudinal research is warranted to examine how perceptions, habits, and learning outcomes by using GenAI tools co-develop over time. These studies may deepen the understanding of these processes. Third, the study is situated within a specific academic context, which may limit the generalizability of the findings to other populations, institutional environments, or cultural settings. Comparative research across disciplines, institutions, and countries would be valuable in assessing the robustness and transferability of the results, particularly in light of contextual differences in technological infrastructure, pedagogical practices, and normative expectations regarding GenAI use. Fourth, there are additional models in the technology literature that could expand and deepen the range of constructs and mechanisms proposed in the current study. Future research could expand on the variables and direct and indirect effects on technology adoption or rejection and provide a more detailed account of how technology-related factors translate into shaping users’ attitudes and learning outcomes. Finally, while this study focuses on key cognitive and behavioral determinants, it does not fully incorporate additional constructs that have recently become prominent in the literature on AI-enabled technologies. Future research should consider integrating factors such as trust in AI, perceived risk, algorithmic transparency, and GenAI literacy, which may further refine and extend existing technology acceptance frameworks.

## 9 Summary

The rapid diffusion of GenAI tools in higher education has transformed the landscape of academic learning, raising critical questions regarding their pedagogical value and impact on students’ learning processes. The current study aimed to examine the relationships between GenAI training, ethical knowledge, experience with GenAI, students’ perception of merits and drawbacks, technological orientation, attitudes toward GenAI tools, and the perceived effectiveness of academic learning using these tools. For this purpose, we utilized a hybrid theoretical framework based on the evolution of acceptance models TAM3 and UTAUT2. This merging comprehensive alignment allowed to identify multidimensional factors influencing students’ intentions and actual usage of GenAI tools. The results of the study demonstrate that technology-related antecedents play a central role in shaping students’ attitudes and their learning effectiveness. The core findings illuminate the centrality of students’ direct experience with GenAI, the importance of institutional training on these tools, the mixed opinions regarding ethical guidelines, and the gap between perceived benefits and concerns arising from their use. From a theoretical perspective, these relationships align with the technology adoption frameworks of the TAM3 and the UTAUT2, which emphasize that perceptions of usefulness, effort, and contextual experience shape both attitudes and behavioral outcomes. By moving beyond a purely utilitarian perspective, the study highlights that the effectiveness of GenAI in academic contexts is not solely a function of perceived usefulness or ease of use but emerges from a complex of cognitive, experiential, and normative factors. Consequently, the study provides empirical support for extending traditional TAMs to account for the distinctive characteristics of GenAI-enabled learning environments.

Beyond its empirical contributions, this research offers important theoretical and practical implications for the evolving landscape of higher education. Theoretically, it underscores the need to conceptualize technology acceptance as a dynamic and context-sensitive process, particularly in the case of GenAI, where issues of ethics in practice, cognitive risks, and user competence are increasingly salient. Practically, the findings suggest that institutions seeking to leverage GenAI for learning enhancement should invest not only in access to technological tools but also in structured training, ethical guidance, and the development of students’ technological orientation. Taken together, the study contributes to a more nuanced understanding of how GenAI technologies can be meaningfully integrated into academic learning and lays the groundwork for future research aimed at refining and extending existing theoretical models in light of rapidly evolving super-smart systems. Ultimately, as GenAI technologies continue to redefine the boundaries of human knowledge production, advancing theoretically grounded and empirically robust models of its adoption is essential for ensuring that GenAI properly integrates into academic systems properly. As higher education institutions will increase the integration of GenAI tools into teaching and learning, translating theoretical insights into actionable pedagogical strategies will be crucial for realizing their full tech-educational potential.

## Data Availability

The original contributions presented in the study are included in the article/Supplementary material, further inquiries can be directed to the corresponding author.
