# Supplementary material for: A multidimensional analysis of generative AI adoption in academia: synthesizing TAM3 and UTAUT2 frameworks to predict students’ attitudes and learning effectiveness
Source: Front Artif Intell. 2026 Oct 7;9:1897946. doi: 10.3389/frai.2026.1897946 (PMC13683649; doi:10.3389/frai.2026.1897946)
Supplement: Supplementary file 1 [file Supplementary_file_1.docx]

**Appendix A. Indicators of students' attitudes, learning effectiveness, training, and ethical knowledge tested via GenAI questionnaire**

| **Sub-scale** | **Questions** | **Cronbach α** |
| --- | --- | --- |
| Formal Training | Have you received training from the academic institution on how to use generative artificial intelligence tools? | 0.72 |
|  | Do you think the training on generative artificial intelligence tools by the academic institution was comprehensive? |  |
|  | Was the training on generative artificial intelligence tools by the academic institution tailored to your academic needs? |  |
|  | Do you think the training on generative artificial intelligence tools by the academic institution makes learning in academia more efficient? |  |
|  | Do you think the training on generative artificial intelligence tools by the academic institution is necessary as part of preparation for the job market? |  |
|  | As part of the training from the academic institution on generative artificial intelligence tools, did you receive explanations and instructions on how to use this technology ethically? |  |
|  | If you have not been trained, do you think you are using this technology ethically? |  |
| Ethical knowledge | Do you know that generative artificial intelligence tools may use your personal information? | 0.69 |
|  | Do you take personal responsibility for the content created by generative artificial intelligence? |  |
|  | Do you think using generative artificial intelligence tools to create original content for academic papers constitutes academic fraud? |  |
|  | Are you aware of the fact that generative artificial intelligence tools also provide information that is inaccurate or unreliable? |  |
|  | Do you know that generative artificial intelligence tools can violate copyright and user privacy? |  |
|  | Are you aware of the fact that generative artificial intelligence tools can produce content that constitutes discrimination or incitement? |  |
| Attitudes | I enjoy using generative artificial intelligence tools in my studies | 0.95 |
|  | Using generative artificial intelligence tools in my studies is an enjoyable experience |  |
|  | Using generative artificial intelligence tools helps me a lot in my academic studies |  |
|  | Overall, I am satisfied with the learning process using generative artificial intelligence tools |  |
|  | I am interested in expanding my use of generative artificial intelligence tools to other areas as well |  |
|  | Using generative artificial intelligence tools in my studies is innovative and interesting |  |
|  | Using generative artificial intelligence tools is a challenging learning experience |  |
|  | I would like to see more generative artificial intelligence tools incorporated as part of the degree curriculum |  |
|  | I would recommend to my friends that they use generative artificial intelligence tools in their studies |  |
|  | It is quite easy and simple for me to use generative artificial intelligence tools |  |
|  | I am comfortable using generative artificial intelligence tools |  |
|  | In general, I am satisfied with using generative artificial intelligence tools |  |
|  | I think that using generative artificial intelligence tools is suitable for academic learning |  |
|  | I feel quite proficient in using generative artificial intelligence tools |  |
|  | I would like to use new generative artificial intelligence tools in the future |  |
| Effectiveness- skills | By using generative artificial intelligence tools, I acquire new ways of learning | 0.91 |
|  | Using generative artificial intelligence tools improves my learning skills |  |
|  | Using generative artificial intelligence tools helps me develop academic learning abilities |  |
|  | Using generative artificial intelligence tools helps me develop self-learning ability |  |
|  | Using generative artificial intelligence tools improves my thinking skills |  |
| Effectiveness- motivation | Using generative artificial intelligence tools increases my interest in the subjects studied in courses | 0.86 |
|  | Using generative artificial intelligence tools contributes to my motivation to learn |  |
|  | I invest a lot of time in learning how to use generative artificial intelligence |  |
|  | Using generative artificial intelligence tools motivates me to study the course materials in more depth |  |
|  | The possibility of practicing and receiving examples, illustrations, and recommendations from generative artificial intelligence tools contributes to my motivation to learn more meaningfully |  |
| Effectiveness- benefits | Using generative artificial intelligence tools helps me develop confidence in my academic studies | 0.91 |
|  | Using generative artificial intelligence tools streamlines my learning processes in academic courses |  |
|  | I use generative artificial intelligence tools a lot in my academic studies |  |
|  | Overall, I learn well with generative artificial intelligence tools |  |
|  | Learning with generative artificial intelligence tools helps me succeed better in my academic studies |  |
| Effectiveness- understanding | Using generative artificial intelligence tools helps me better understand the course materials | 0.85 |
|  | Using generative artificial intelligence tools helps me better understand complex concepts, solve problems, perform tasks, and develop new perspectives |  |
|  | I think that I would understand the course topics better if they taught me how to use generative artificial intelligence tools in the lessons |  |
|  | Overall, I have a good understanding of the queries results are received by generative artificial intelligence tools |  |
|  | The feedback received by generative artificial intelligence tools helps me better understand the course content |  |
